# Cynanchum bungei Decne-derived extracellular vesicles alleviate cognitive impairment and pathological damage in Alzheimer’s disease

**DOI:** 10.3389/fncel.2026.1798965

**Published:** 2026-04-10

**Authors:** Rui Hong, Jingjing Han, Fuxing Dong, Umm-E Kalsoom, Cong Cao, Aihua Zhou, Qin Wu, Xuebin Qu

**Affiliations:** 1 Department of Basic Medical Science, Jiangsu Medical College, Yancheng, Jiangsu, China; 2 School of Life Science and Technology, ShanghaiTech University, Shanghai, China; 3 Public Experimental Research Center of Xuzhou Medical University, Xuzhou, Jiangsu, China; 4 The Fourth People’s Hospital of Yancheng, Yancheng, Jiangsu, China

**Keywords:** Alzheimer’s disease, *Cynanchum bungei* Decne, extracellular vesicles, NUPR1, oxidative stress

## Abstract

**Introduction:**

*Cynanchum bungei* Decne (CB) is known for its therapeutic benefits for neurodegenerative conditions as anti-inflammatory, antioxidant, and barrier significantly limits their potential advantages. Given the ability of crossing the barrier with minimal toxicity, extracellular vesicles derived from CB (CB-EVs) were utilized as an innovative approach to mitigate Alzheimer’s disease (AD).

**Methods:**

CB-EVs were isolated using gradient ultracentrifugation and identified via TEM imaging, nanoparticle tracking analysis, marker identification, and *in vivo* imaging system. Ten-month-old triple transgenic AD (3xTg-AD) mice received intravenous administration of CB-EVs at doses of 10 or 20 mg/kg every 3 days for the cognitive and pathological assessments. The human APP Swedish mutation transgenic SH-SY5Y cells were constructed as Aβ-induced neural damage model, and different concentrations of CB-EVs were added into medium to analyze its roles on cell viability, transcriptome changes, oxidative stress, and mitochondrial damage.

**Results:**

CB-EVs exhibited standard morphological and molecular traits, accumulating in the cerebral cortex and hippocampus. Two months of CB-EVs treatment alleviated cognitive impairments, diminished Aβ plaque, reduced Tau protein hyperphosphorylation, and lessened neuronal loss in 3xTg-AD mice. In transgenic SH-SY5Y cells, CB-EVs improved cell viability, enhanced superoxide dismutase activity, downregulated oxidative stress related NUPR1 and CHOP expression, decreased reactive oxygen species, lipid peroxidation, and malondialdehyde levels, reduced mitochondrial damage.

**Conclusion:**

These results demonstrated that CB-EVs could protect neurons from oxidative stress, attenuate cognitive impairment and pathological damage in AD.

## Graphical Abstract

**Figure:**
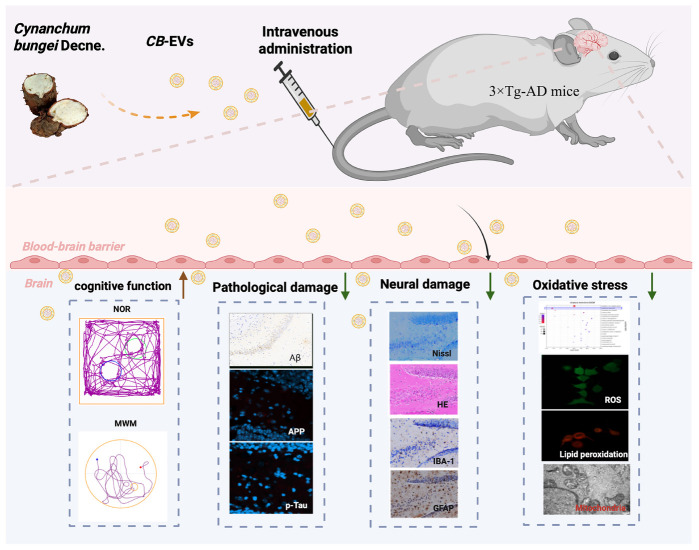
*Cynanchum bungei* Decne. derived extracellular vesicles (*CB*-EVs) were used as a novel strategy for AD alleviation to mitigate cognitive disorders, reduce Aβ plaque, downregulate protein Tau hyperphosphorylation, enhance neuron viability, relieve oxidative stress and alleviate mitochondria damage.

## Introduction

Alzheimer’s disease (AD) progressively affects the nervous system, leading to memory deterioration, cognitive challenges, language problems, and changes in behavior (Knopman et al., 2021). The Alzheimer’s Association reported that more than 50 million people worldwide were living with dementia in 2018, and it was expected that this figure would triple by 2050, with a new case emerging every three seconds. Despite a lot of effort has been put into the research of mechanisms and therapies, the exact causes of AD remain unclear, even there are still no effective treatments (Leng and Edison, 2021). The amyloid and Tau hypotheses propose that excessive β-amyloid (Aβ) peptides initiate Tau hyperphosphorylation, which disrupts synaptic function, promotes inflammation and oxidative damage, and results in Aβ plaques and neurofibrillary tangles. Unfortunately, numerous clinical trials targeting these pathways have largely failed (Rodriguez et al., 2021; Wojtunik-Kulesza et al., 2023), underscoring the need for deeper exploration of AD pathogenesis and alternative treatment strategies.

Growing evidence highlights oxidative stress as a key contributor to AD (Umar et al., 2024). AD progression is tightly linked to excessive reactive oxygen species (ROS) production (Park et al., 2021), weakened antioxidant systems, mitochondrial dysfunction, and calcium imbalance (Tapias et al., 2023), cellular injury and apoptosis (Monteiro et al., 2023). As aging progresses, the body’s capacity to maintain oxidative balance declines, contributing to further neurodegenerative disorders. Research suggests that oxidative damage contributes to Tau hyperphosphorylation and Aβ_25–35_ formation, worsening disease pathology (Liu et al., 2023). Considering the significant impact of oxidative stress in AD pathogenesis, antioxidant-based therapeutic strategies appear promising. Clinical studies indicate that antioxidants like vitamins E and C can slow dementia progression by neutralizing free radicals and reducing oxidative damage (Tabet et al., 2002). In addition, many natural compounds have shown potential for treating AD via resisting inflammatory response, ameliorating oxidative stress, regulating Aβ metabolism, preventing neuronal apoptosis, and protecting against scopolamine-induced aging (Liu et al., 2023; Mir et al., 2021; Shah et al., 2025).

*Cynanchum bungei* Decne. (CB) is a traditional medicinal plant abundant in essential nutrients and bioactive compounds, contributing to its anti-inflammatory, antioxidant, immunomodulatory, and neuroprotective properties (Chen et al., 2019). Studies show that the C21 steroidal glycosides Cynsaccatol Q and Saccatol K from CB provide neuroprotection against oxidative damage by lowering intracellular ROS and Ca2^+^ levels, thus preventing H_2_O_2_-induced apoptosis (Zhang et al., 2021). CB enhances the survival of SH-SY5Y cells exposed to Aβ oligomer, demonstrating a protective effect. However, the restricted capacity of *CB* and its extract to penetrate the blood–brain barrier (BBB) *in vivo* significantly limits their therapeutic potential.

Plant-derived extracellular vesicles (PDEVs), measuring 30–200 nm, transport genetic material and proteins, enabling intercellular communication and signal transduction (Kalluri and LeBleu, 2020). PDEVs, due to their diminutive size, can pass through the BBB, which positions them as promising candidates for translational uses (Ramos-Zaldivar et al., 2022). In this study, EVs were effectively isolated and purified from CB following established characterization criteria. Behavioral and pathological assessments demonstrated that CB-EVs provided significant neuroprotection in triple transgenic AD (3 × Tg-AD) mice.

## Materials and methods

### Isolation of CB-EVs

Fresh CB was juiced, and then subjected to sequential centrifugation. The supernatant was subjected to ultracentrifugation at 150,000 *g* (Beckman Coulter Life Sciences, Indianapolis, United States). The pellets were resuspended in phosphate buffered saline (PBS) and subjected to ultracentrifugation for 90 min in a gradient sucrose solution. The band between 30% and 45% sucrose layer was collected, ultracentrifuged and filtrated through a 0.22 μm filter (#SLGV004SL, Millipore) to obtain sterile CB-EVs. Protein quantification of CB-EVs was performed using the BCA assay kit (Beyotime, #P001). In some trials, purified CB-EVs were heat-inactivated by incubation at 95 °C for 10 min.

### Animals

3xTg-AD mice, featuring human mutations APPswe, PS1M126V, and TauP301L, along with age-matched wild-type (WT) mice, were sourced from HFK Bioscience Co., Ltd. (Beijing, China; license number SYXK 2018-0008). Due to significant neuropathological variability in 3xTg-AD males, this study included only female mice, consistent with prior research (Li et al., 2025). Mice were provided with a standard diet and distilled water, following a 12-h light-dark cycle. The Ethics Committee of Jiangsu Medical College (XMLL-2022-056) approved the experiments. The 3xTg-AD mice were randomly assigned to three groups: the vehicle-treated AD model group (saline only), the 10 mg/kg CB-EVs-treated group, and the 20 mg/kg CB-EVs-treated group. The required amount of CB-EVs for each mouse was calculated as the target dose multiplied by body weight, and each mouse was injected with an equal volume of saline containing varying concentrations of CB-EVs. All mice were treated once every 3 days for 2 months. Wild-type mice received intravenous saline on the same schedule served as the control group. The number of mice in each group was calculated based on the preliminary experiment using power calculation, meeting the criteria of *p* = 0.05 and power = 0.8.

### Cell culture

SH-SY5Y cells were maintained in Dulbecco’s Modified Eagle’s Medium (DMEM) (VICMED, China) with 10% fetal bovine serum (FBS, Meilunbio, China) and 1% penicillin/streptomycin (Keygentec, China). Cultures were incubated at 37 °C with 5% CO_2_ in a humidified environment.

### Cell transfection

SH-SY5Y cells were transduced with the lentiviral packaging APP Swedish mutation plasmid (Genepharma, China) at a multiplicity of infection of 10. Cells were subjected to selection with a medium containing 4 μg/ml puromycin after 48 h. One week later, the expression of APP was assessed.

### Nanoparticle tracking analysis

CB-EVs were diluted with PBS and introduced into the analytical cell of nanoparticle tracking analyzer (Zeta View, Bavaria, Germany) to assess the size distribution and quantification of CB-EVs.

### *In vivo* uptake of CB-EVs

The isolated CB-EVs were incubated with the lipophilic fluorescent dye Dil (Molecular Probes, Oregon, United States) for 30 min at room temperature. The Dil-labeled CB-EVs (CB-EVs+Dil) were ultracentrifuged at 150,000 *g* for 90 min, then resuspended in PBS. Mice were intravenously administrated with CB-EVs + Dil or free-Dil, and then imaged by the IVIS^®^ Spectrum system (Perkin Elmer, Massachusetts, United States) at 1, 24, 72, 96 h after administration. Cryosections of mouse brains were prepared and examined with a Leica confocal laser scanning microscope (Germany).

### Morris water maze

The Morris water maze (MWM) setup featured a circular pool with a 5 cm diameter white platform. Following 6 weeks of CB-EVs treatment, the mice underwent pre-training with a visible platform, consisting of 4 trials over 5 days. Spatial learning scores were assessed by recording the escape latency and path length before the mice located the hidden platform. The count of times mice crossed the initial platform location was documented. On the sixth day, the platform was removed, and the crossing times, the swimming distance and time in the target quadrant were recorded by a video analysis system (Any maze, United States).

### Novel object recognition test

For the novel object recognition (NOR) test, mice were exposed to two identical objects for 5 min in a 40 × 40 × 40 cm box. On the third day, a familiar object was replaced with a novel one in the same location. The mice’s behavioral performance was recorded for 5 min, followed by the analysis of both duration and frequency of a mouse’s interactions with each object (Any maze, United States). The object preference index was calculated by comparing the time spent exploring the new object to the total time spent on both the familiar and new objects.

### Hematoxylin-eosin (HE) and Nissl staining

Mice were euthanized and brain tissues were collected and fixed in 10% formalin, and sequentially dehydrated in 70%, 95%, and 100% ethanol, followed by xylene. The brain slides, sectioned into 4 μm after paraffin embedding, were stained with HE. For Nissl staining, the brain slides were immersed in lutidine blue (71044080, Sinopharm, Beijing, China) at 56 °C for 1 h. The slides were examined using an Olympus BX53 optical microscope.

### Thioflavin-S (TS) staining

Cryosections of mouse brains were prepared and immersed in 1% TS solution for 5 min, then soaked in 70% ethanol, rinsed, and sealed. Senile plaques in tissue sections were examined with a Leica confocal laser scanning microscope and analyzed using Image J.

### Cell viability test

Cells were incubated with CCK-8 solution (VICMED, China) for 2 h, followed by recording the absorbance at 405 nm.

### Western blot

Mouse brain tissues, SH-SY5Y cells or *CB*-EVs were homogenized with ice-cold RIPA buffer (#WB3100, NCM Biotech) containing 1% phosphatase and protease inhibitors (#P003, NCM Biotech). The BCA assay kit (Meilunbio, China) was used to measure total protein concentration. Proteins were resolved using SDS-PAGE gels (Key GEN Bio Tech, #KGC4407-50) and subsequently transferred onto 0.22 μm PVDF membranes (Millipore, #00195863). After being blocked, membranes were incubated overnight with the primary antibody at 4 °C. The primary antibodies used were CD63 (1:500, #25682-1-AP, Proteintech), CD9 (1:1000, #20597-1-AP, Proteintech), GM130 (1:50000, #11308-1-AP, Proteintech), GAPDH (1:1000, #AF2819, Beyotime), APP (1:10000, #255241-AP, Proteintech), P-Tau (1:500, #28666-1-AP, Proteintech), PSD-95 (1:5000, #30255-1-AP, Proteintech), Synaptophysin (1:5000, #17785-1-AP, Proteintech), NUPR1 (1:200, #15056-1-AP, Proteintech), CHOP (1:1000, #66741-1-Ig, Proteintech), and β-tubulin (1:1000, #AF2835, Beyotime). Then, the membranes were incubated with secondary antibodies for 1 h at room temperature, and blots were visualized using an ECL detection kit (NCM Biotech, #P10100). Image J software was utilized to analyze the band density.

### Immunohistological staining

Paraffin or cryo-sections were prepared, blocked with goat serum, and incubated overnight at 4 °C with the following primary antibodies: NeuN (1:300, #26975-1-AP, Proteintech), Aβ_1–42_ (1:500, #ab201061, Abcam), Iba-1 (1:300, #019-19741, WAKO), GFAP (1:400, #ab207165, Abcam), APP (1:10000, #255241-AP, Proteintech), P-Tau (1:500, #28666-1-AP, Proteintech), PSD-95 (1:5000, #30255-1-AP, Proteintech), and Synaptophysin (1:5000, #17785-1-AP, Proteintech). A secondary antibody, either enzyme-labeled or fluorescent, was utilized. Images were captured with a Leica confocal laser scanning microscope (Germany) following counterstaining and analyzed using Image J.

### Transcriptomics

Total RNA from SH-SY5Y cells was quantified at Majorbio Biopharm Technology Co., Ltd. in Shanghai, China. Data analysis was conducted using the Majorbio Cloud Platform^1^, a free online resource.

### ROS levels

Reactive oxygen species (ROS) levels were measured using an assay kit (Cat#S0033S) from Beyotime, China. Cells were incubated with a 10 μM DCFH-DA probe in serum-free medium for 30 min at 37 °C.Samples were examined with a ZEISS LSM900 laser confocal microscope, and fluorescence intensity was analyzed using Image J software.

### Malondialdehyde (MDA) measurement

Samples were prepared following the MDA detection kit instructions (#S0131S, Beyotime, China), and absorbance was measured at 532 nm. The MDA concentration was determined using the specified formula from the instructions.

### Lipid oxidation detection

Cells were incubated at 37 °C for 20 min in a 2 μm solution of BODIPY 581/591 C11 (#S0043S, Beyotime, China). The oxidized probe emitted at 488–510 nm (green), whereas the non-oxidized probe emitted at 581–591 nm (red). Images were captured with a ZEISS LSM900 confocal microscope (Germany). Image J was used to quantify fluorescence intensity.

### Detection of superoxide dismutase (SOD) levels

Superoxide dismutase levels were measured using a test kit (Cat#S101S, Beyotime, China) following the instructions. The absorbance was determined at 450 nm.

### Enzyme-linked immunosorbent assay (ELISA)

Soluble Aβ concentrations were measured using ELISA kits (#JL11386, Jonln bio., China) following the manufacturer’s protocols. Absorbance at 450 nm was measured using a microplate reader for analysis.

### Transmission electron microscopy (TEM)

*Cynanchum bungei* Decne-extracellular vesicles were adsorbed to a carbon-coated grid for 1 min, followed by washing with one drop water and stained for 15 s with 1% uranyl acetate.SH-SY5Y cells were fixed with 2.5% glutaraldehyde, processed through dehydration, infiltration, embedding, and sectioning to obtain ultrathin sections, and stained with uranyl acetate and lead citrate. Images were acquired with TEM (JEM1400, Japan) for analysis.

### Statistical analysis

All experiments were conducted in a double-blind manner. Statistical analyses were conducted using Prism 9.0. Data are expressed as mean ± SEM. One-way analysis of variance (ANOVA) followed by Bonferroni’s *post-hoc* test was used for group comparisons. MWM escape latencies during the training days were evaluated by two-way repeated-measures ANOVA. A *p*-value below 0.05 was deemed statistically significant.

## Results

### Isolation and characterization of CB-EVs

**FIGURE 1:**
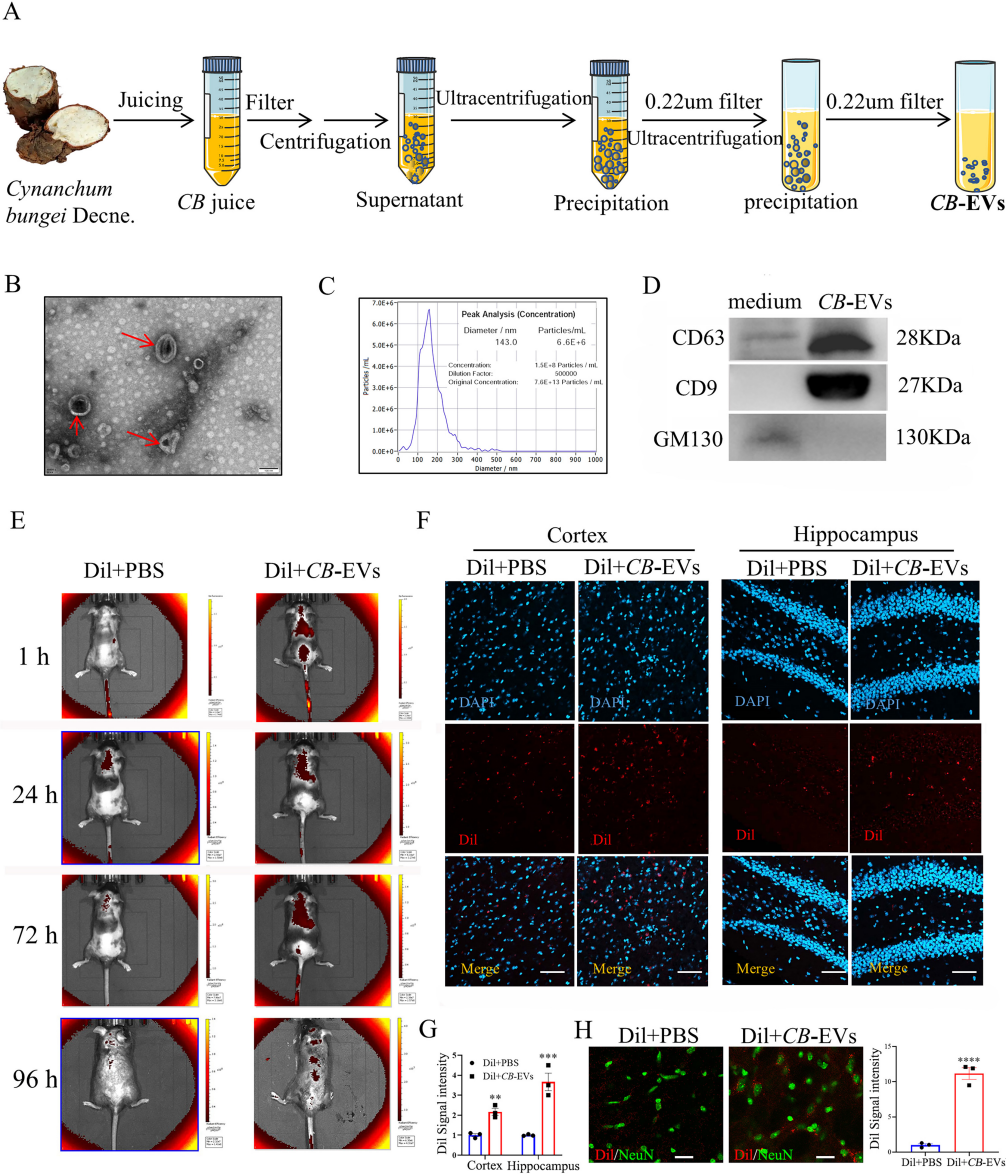
*Cynanchum bungei* Decne-extracellular vesicles (CB-EVs) were isolated and identified. **(A)** Isolation process of CB-EVs. **(B)** TEM image of CB-EVs (red arrow). Scale bar, 200 nm. **(C)** Size distribution and doze of CB-EVs. **(D)** The expressions of CD63, CD9, and GM130. **(E)** The location of Dil in mice by *in vivo* imaging system after intravenous administration. **(F,G)** Representative Dil fluorescence images and fluorescence intensity quantification on the brain slices from mice 72 h after administration. Scale bar, 100 μm. **(H)** Images and quantification of Dil co-staining with NeuN on the brain slices. Scale bar, 50 μm. Data represented as mean ± SEM, **P < 0.01, ***P < 0.001, ****P < 0.0001.

*Cynanchum bungei* Decne-extracellular vesicles were isolated via gradient ultracentrifugation (Figure 1A), and characterized by irregular membrane-enclosed structure (Figure 1B) in an average diameter of 150 nm (Figure 1C), positive expression of CD63 and CD9, and negative for GM130 (Figure 1D). To examine *in vivo* CB-EVs uptake, Dil-labeled CB-EVs were intravenously administered to mice. *In vivo* imaging revealed strong red fluorescence signals indicating CB-EVs accumulation in the brain, persisting up to 72 h post-administration and then began to decrease (Figure 1E). Fluorescence image of brain tissue sections revealed that Dil labeled CB-EVs aggregated in cerebral cortex and hippocampus (Figures 1F, G), and co-localized with neurons (Figure 1H).

### CB-EVs improved cognitive impairment in 3xTg-AD mice

**FIGURE 2:**
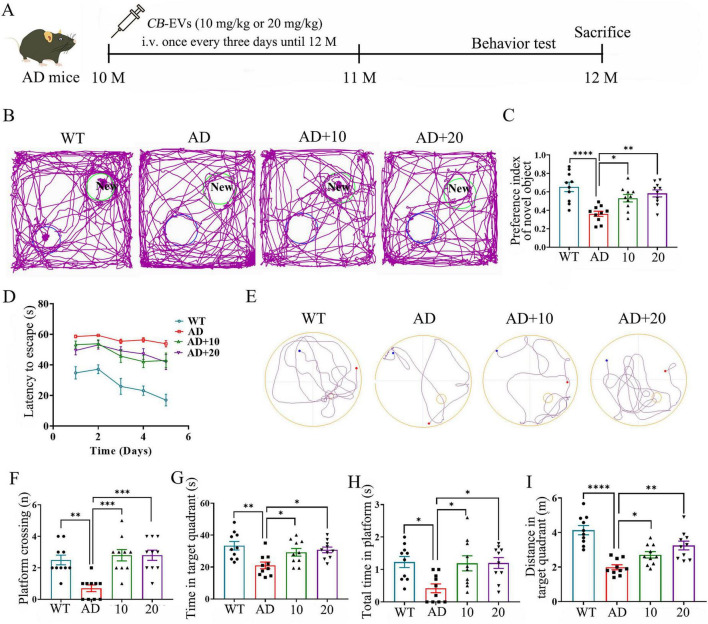
*Cynanchum bungei* Decne-extracellular vesicles (CB-EVs) improve cognitive impairment in 3xTg-AD mice. **(A)** Timeline of CB-EVs treatment and behavior test. **(B)** Representative traces of mice in NOR test. AD+10, 3xTg-AD mice intravenous administrated with 10 mg/kg *CB*-EVs. AD+20, 3xTg-AD mice intravenous administrated with 20 mg/kg CB-EVs. **(C)** Preference index of novel objective in the NOR test. **(D)** Escape latencies during the training days. **(E)** Representative traces of mice in MWM test at day 6. **(F)** Crossing number over the platform site in test at day 6. **(G,H)** Time spent in platform and target quadrant. **(I)** Distance in the target quadrant. *n* = 10 per group for each trial. Data represented as mean ± SEM, *P < 0.05, **P < 0.01, ***P < 0.001, ****P < 0.0001.

Ten-month-old 3xTg-AD mice received intravenous administration of CB-EVs at doses of 10 or 20 mg/kg body weight over 2 months, with behavioral tests performed during the last month (Figure 2A). The NOR test assessed cognitive ability by leveraging rodents’ natural preference for exploring novel objects (NO) over familiar ones. The findings indicated that 3xTg-AD mice spent less time on NO, while CB-EVs treated 3xTg-AD mice explored NO more frequently (Figures 2B, C). In MWM test, during the 5-day training period, all mice had a gradually decreasing escape latency (Figure 2D). CB-EVs treatment reduced the significantly increased escape latency observed in 3xTg-AD mice compared to WT mice (Figure 2D). On the sixth day, after the platform was removed, CB-EVs treated 3xTg-AD mice crossed the former platform location more frequently and spent more time in the target quadrant compared to untreated 3xTg-AD mice (Figures 2E–I). These data showed that both 10 and 20 mg/kg CB-EVs effectively improved the severe decline in cognitive function of 3xTg-AD mice, but there was no significant difference between mice treated with two different doses of CB-EVs (Figure 2).

### CB-EVs reduced pathological damage in 3xTg-AD mice

**FIGURE 3:**
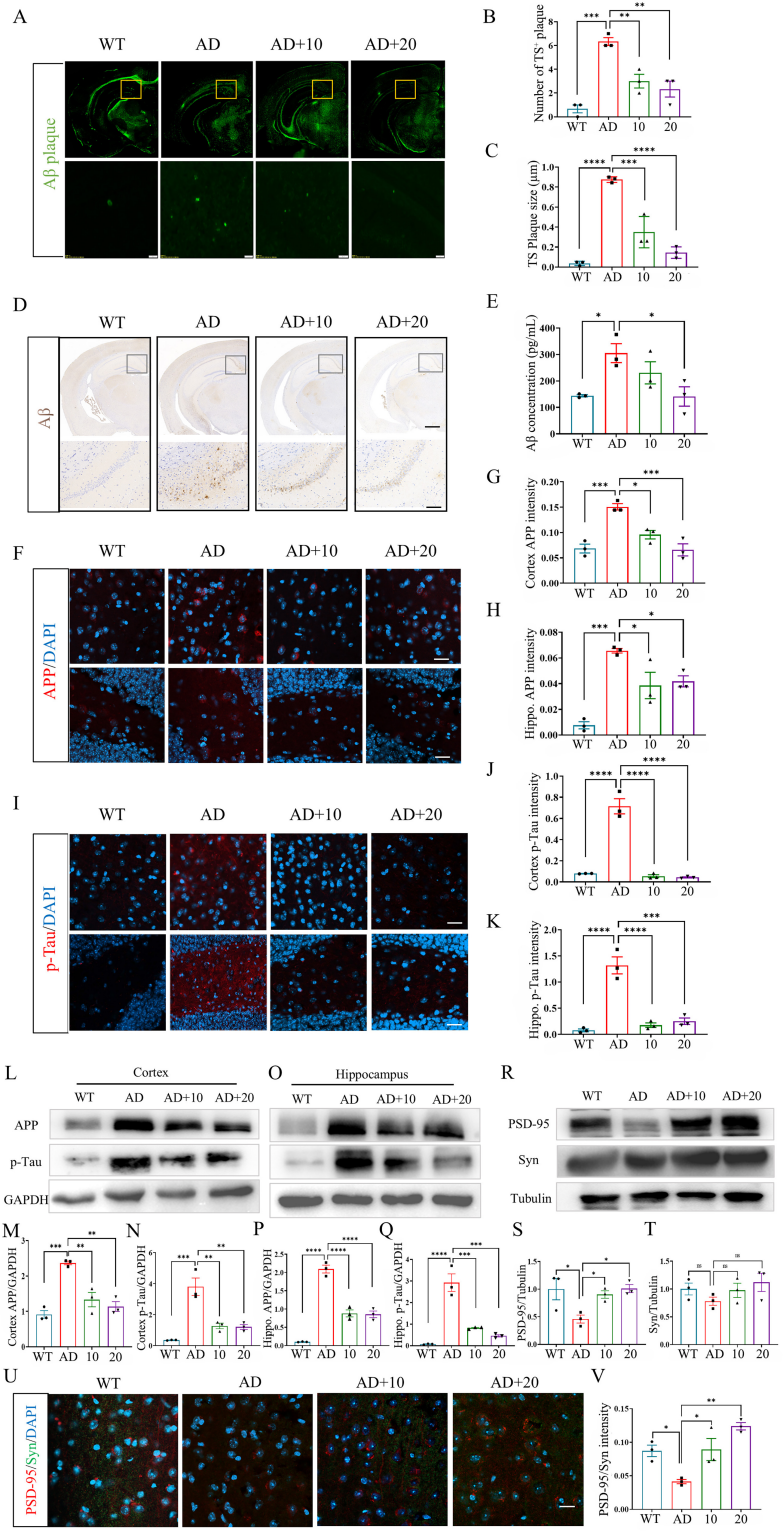
*Cynanchum bungei* Decne-extracellular vesicles (CB-EVs) reduce Aβ plaques and hyperphosphorylated Tau in 3xTg-AD mice. **(A)** Representative images of Aβ plaques by TS staining. The image in the box is enlarged below. **(B,C)** Statistical analysis of TS^+^ plaques. **(D**) Immunohistochemical images of Aβ in brain. **(E)** ELISA of Aβ concentration. **(F–K)** Immunofluorescence analysis of APP and p-Tau in the cortex and hippocampus. **(L–T)** Western blot analysis of APP, p-Tau in the cortex and hippocampus, and Synaptophysin (Syn), PSD95 in the hippocampus. **(U,V)** Immunofluorescence analysis of Synaptophysin and PSD95 in the hippocampus. Data represented as mean ± SEM, *P < 0.05, **P < 0.01, ***P < 0.001, ****P < 0.0001.

To evaluate the impact of CB-EVs on pathological changes in 3xTg-AD mice, Aβ plaques in the brain were analyzed using TS-staining and immunohistology. The findings indicated that CB-EVs reduced both the quantity and size of plaques in the cortex and hippocampus of 3xTg-AD mice (Figures 3A–D). Treatment with CB-EVs significantly reduced the concentration of soluble Aβ in the brain (Figure 3E). In addition, CB-EVs treatment significantly reduced the elevated levels of amyloid precursor protein (APP) and phosphorylated Tau (p-Tau) in the brains of 3xTg-AD mice, as demonstrated by both the data in Figures 3F–K and the western blot results in Figures 3L–Q. Moreover, the levels of neuronal synaptic protein, PSD95, was measured and showed an obvious decrease in 3xTg-AD mice, while after 2 months of CB-EVs treatment, its expression was remarkably enhanced (Figures 3R–V). Compared with 10 mg/kg CB-EVs treated group, the 20 mg/kg CB-EVs treated AD mice did not exhibit significant differences in the pathological indicators (Figure 3). These data demonstrated that CB-EVs treated 3xTg-AD mice displayed relieved pathological symptoms.

### CB-EVs mitigated neural damage in hippocampus and cortex of 3xTg-AD mice

**FIGURE 4:**
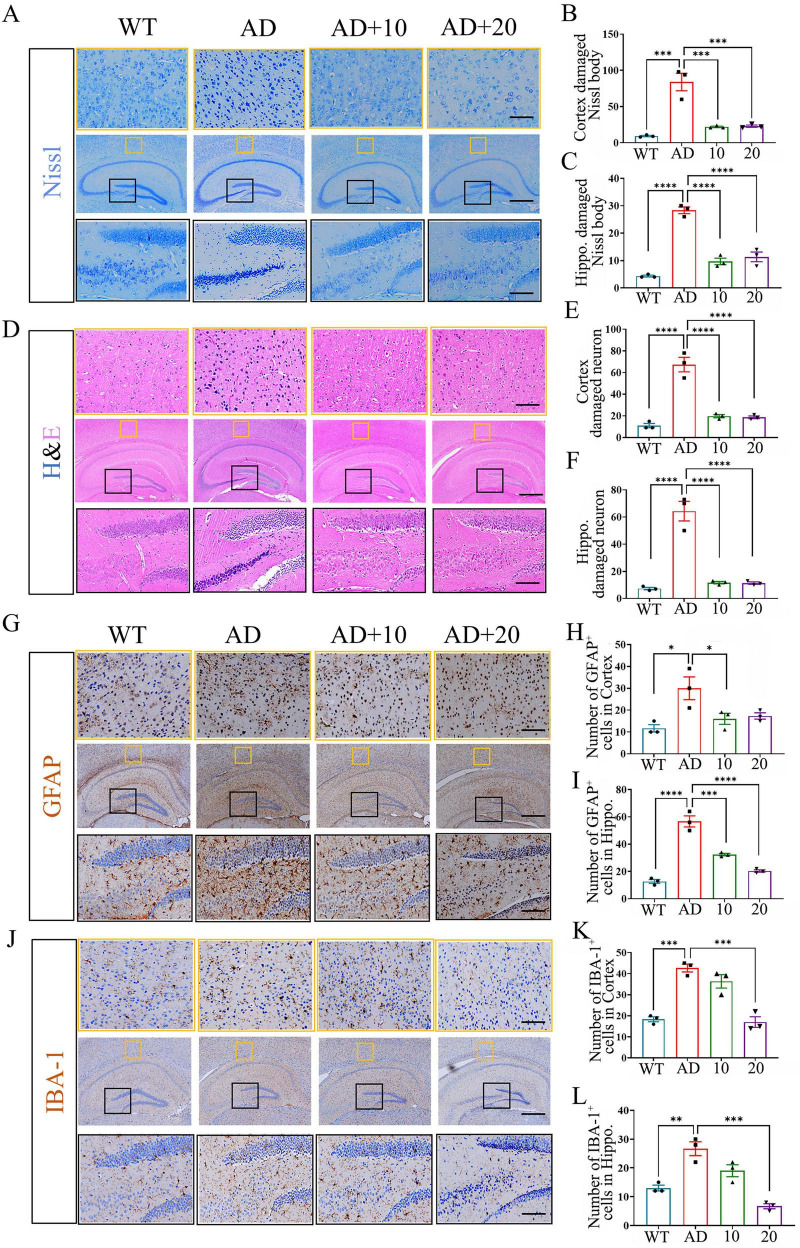
*Cynanchum bungei* Decne-extracellular vesicles (CB-EVs) alleviate cytopathology in hippocampus and cortex of 3xTg-AD mice. **(A–C)** Staining and quantification analysis of Nissl bodies in hippocampus and cortex. **(D–F)** HE staining and analysis of damaged neurons in hippocampus and cortex. **(G–L)** Immunohistochemical staining and quantification analysis of GFAP and IBA-1 positive cells in hippocampus and cortex. Data represented as SH-SY5Y cells. NC, negative control. mean ± SEM, *P < 0.05, **P < 0.01, ***P < 0.001, ****P < 0.0001.

Nissl and HE staining of the hippocampus and cortex showed that 3xTg-AD mice presented disordered and damaged Nissl bodies as well as chromatic agglutination and karyopyknosis, which was improved after CB-EVs treatment (Figures 4A–F). Significant upregulation of GFAP and IBA-1 expression in the cerebral cortex and hippocampus of 3xTg-AD mice indicated increased reactive astrocytes and microglia. CB-EVs significantly decreased the count of GFAP and IBA-1 positive cells in the cortex and hippocampus, suggesting their suppressive effect on astrocyte and microglia activation in 3xTg-AD mice (Figures 4G–L). However, similar to the previous results, the 20 mg/kg CB-EVs treatment did not show better effects compared to the 10 mg/kg group.

### CB-EVs protected hippocampal neurons from Aβ damage

**FIGURE 5:**
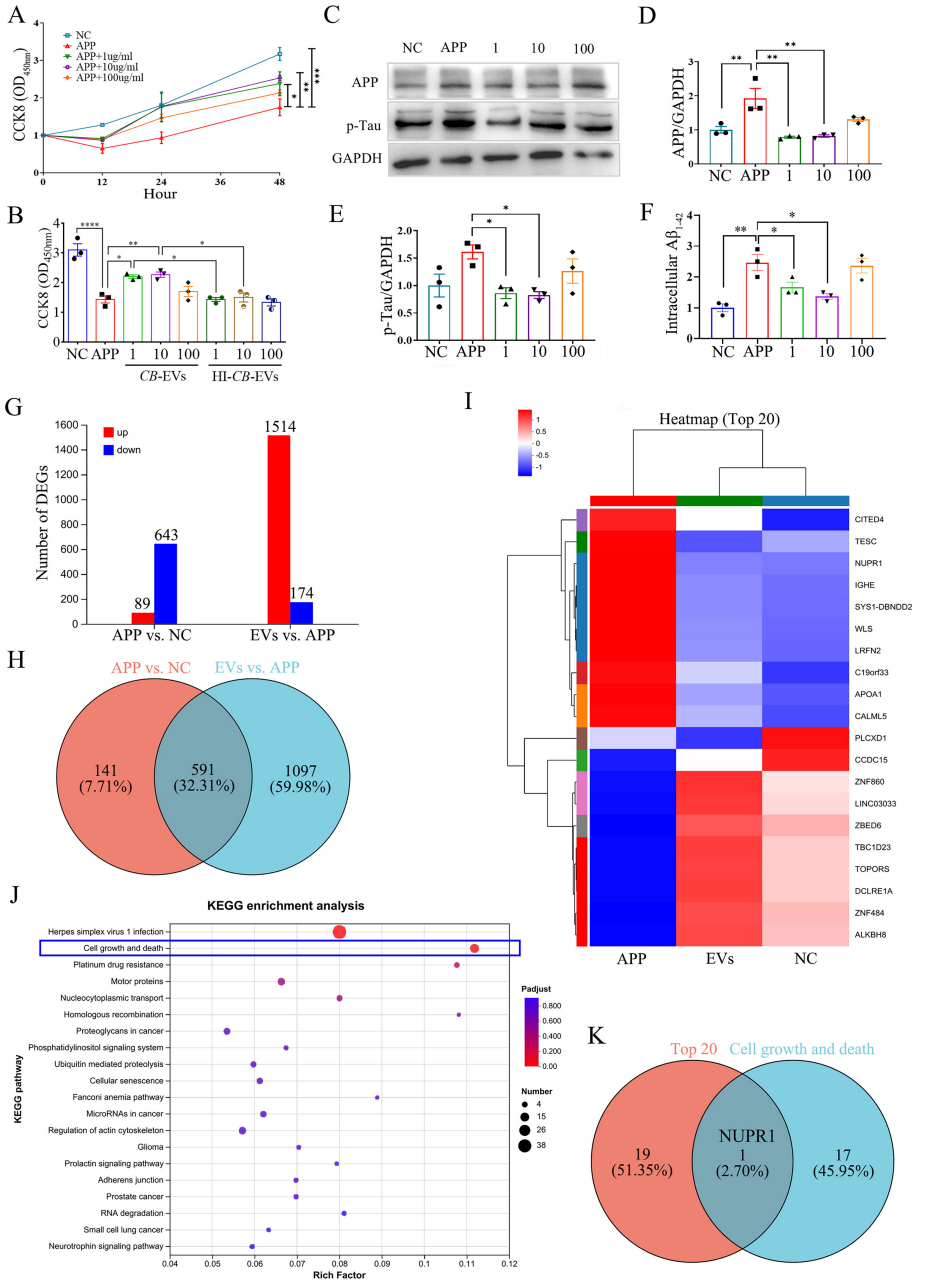
*Cynanchum bungei* Decne-extracellular vesicles (CB-EVs) protect neurons from Aβ damage. **(A)** The relative viability of APP, APP-overexpressed SH-SY5Y cell. Different doses of CB-EVs (1, 10, 100 μg/ml) were added into medium. **(B)** The relative viability of SH-SY5Y cells after 48 h treatment of CB-EVs or heat-inactivated (HI)-CB-EVs. **(C–E)** Western blot analysis of APP and p-Tau in SH-SY5Y cells. **(F)** ELISA of Aβ concentration multiple. **(G,H)** Number of differential expressed genes (DEGs) among NC, APP and 1 μg/ml CB-EVs (EVs) treated SH-SY5Y cells by transcriptome sequencing analysis. **(I)** Clustered heat map of top 20 DEGs among NC, APP and EVs groups. **(J)** KEGG enrichment analysis of DEGs. **(K)** Venn diagram shows the shared gene (NUPR1) of top 20 DEGs and cell growth and death related DEGs. Data represented as mean ± SEM, *P < 0.05, **P < 0.01, ***P < 0.001, ****P < 0.0001.

The Aβ-induced neural damage model was constructed by overexpressing APP in SH-SY5Y cells. CCK8 assay displayed that the viability of cells was markedly increased after the treatment of 1 and 10 μg/ml *CB*-EVs (Figures 5A, B). To validate whether the observed effects were due to biologically active components, CB-EVs were heat-inactivated prior to cell treatment. After 48 h treatment, the viability of APP overexpressed cells exposed to heat-inactivated CB-EVs was nearly identical to that of the PBS-treated cells (Figure 5B). Changes in intracellular protein levels suggested that an appropriate concentration (1 and 10 μg/ml) of CB-EVs significantly decreased the levels of APP, p-Tau and Aβ in APP overexpressed SH-SY5Y cells (Figures 5C–F).

Transcriptome sequencing analysis was performed among negative vector transfected SH-SY5Y cells (NC), APP overexpressed SH-SY5Y cells (APP) and CB-EVs treated APP cells (EVs), and 591 differentially expressed genes (DEGs) were screened out (Figures 5G, H). The top 20 DEGs indicated that CB-EVs treated APP cells exhibited a transcriptional profile similar to NC cells (Figure 5I). KEGG enrichment analysis of the 591 DEGs revealed that genes related to cell growth and death exhibited the highest Rich Factor, indicating the most significant difference (Figure 5J). Then, the shared gene, NUPR1, between top 20 DEGs and cell growth and death related DEGs was screened (Figure 5K), suggesting a potential target for *CB*-EVs to play neuroprotective effects.

### CB-EVs ameliorated oxidative stress damage in neurons

**FIGURE 6:**
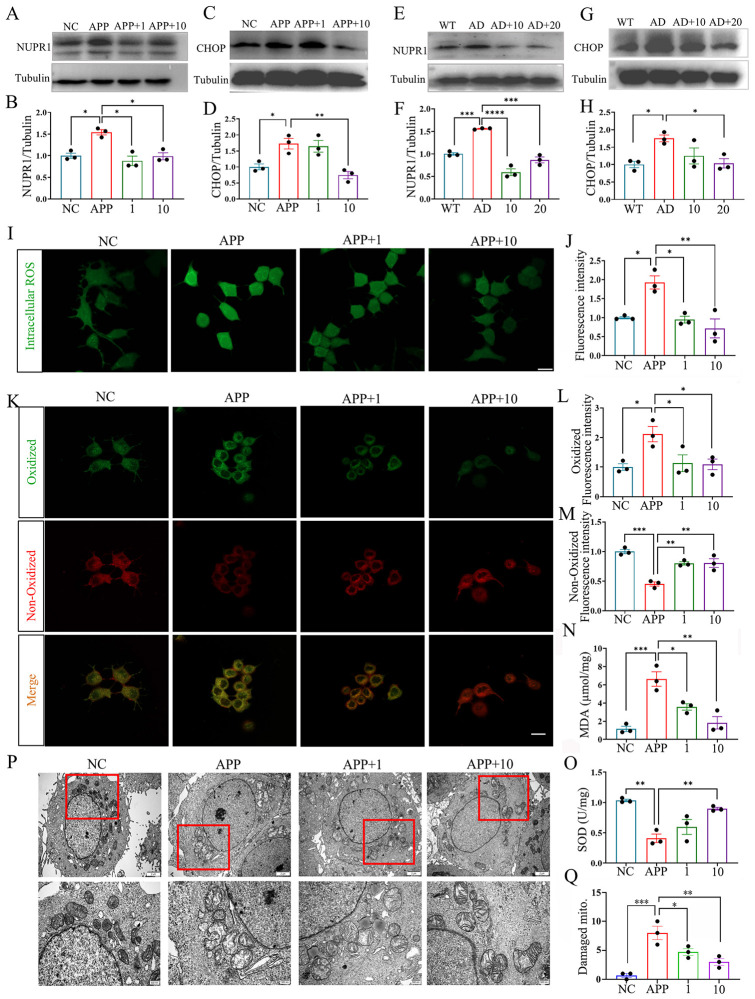
*Cynanchum bungei* Decne-extracellular vesicles (CB-EVs) ameliorate oxidative stress in neurons. **(A–H)** Western blot analysis of NUPR1 and CHOP in SH-SY5Y cells and brain. **(I,J)** ROS detection and quantification analysis by fluorescence staining. **(K–M)** C11-BODIPY staining and quantification analysis of lipid peroxidation. The oxidized probe is colored green, and the non-oxidized probe is red. **(N)** Measurement of MDA content in SH-SY5Y cells. **(O)** Analysis of SOD activity in SH-SY5Y cells. **(P,Q)** Observation and quantification of damaged mitochondrial by TEM. Data represented as mean ± SEM, *P < 0.05, **P < 0.01, ***P < 0.001, ****P < 0.0001.

Given the pivotal roles of NUPR1 and its interacting protein CHOP in modulating oxidative stress, this study explored the impact of CB-EVs on oxidative stress regulation in AD. CB-EVs administration significantly reduced NUPR1 and CHOP expression in both 3xTg-AD mice and APP cells (Figures 6A–H). Then, assessments of reactive oxygen species (ROS), lipid peroxidation, and malondialdehyde (MDA) revealed that CB-EVs significantly decreased ROS and lipid peroxidation levels in APP-damaged SH-SY5Y cells to normal levels (Figures 6I–N). Correspondingly, the activity of superoxide dismutase (SOD) was obviously increased after CB-EVs treatment (Figure 6O). TEM images revealed that APP-damaged neurons showed specific mitochondrial injuries, such as reduced or absent cristae, vacuolization, and partial outer membrane degradation. While in the CB-EVs treated cells, the mitochondrial cristae were preserved, and the membranes remained relatively intact (Figures 6P, Q). In Figure 6, these findings indicated that both 1 and 10 μg/ml CB-EVs could mitigate APP-induced oxidative stress in neurons, but there was no significant difference between them.

## Discussion

Recently, the use of medicinal herbs to prevent neurodegenerative diseases is increasing (Fazmiya et al., 2022). Compared to synthetic drug that target a single site, natural compounds are considered safer and comprises numerous active components targeting multiple sites concurrently to create a synergistic effect in AD treatment (Chen et al., 2024). For instance, *Polygonum multiflorum* Thunb., *Acorus tatarinowii* Schott (Huang et al., 2024), and *Polygala tenuifolia* Willd. (Park et al., 2019) have shown effectiveness in reducing AD-related pathological damage and enhancing cognitive function. Later studies identified Macamides as the key compounds offering neuroprotection by lowering intracellular reactive oxygen species (ROS) and calcium ion (Ca^2+^) levels, thus inhibiting cell apoptosis (Zhang et al., 2021). In addition, CB has been shown to increase SOD and glutathione (GSH) levels to alleviate oxidative stress and achieve intracellular antioxidant effects (Hu et al., 2021). Nevertheless, the exact molecular mechanisms through which CB treats AD are still not fully understood.

Plant-derived extracellular vesicles possess anticancer, anti-inflammatory, antioxidant, and regenerative properties because of containing many types of biologically active components, such as lipids, proteins, nucleic acids, and secondary metabolites (Lo et al., 2024). It has been shown that Aloe vera-coated curcumin encapsulated nanoparticles have therapeutic potential in an Alzheimer-induced mice model (Sharma et al., 2024). Even, clinical trials concentrating on PDEVs from curcumin, ginger, aloe, and grape are currently underway (Lian et al., 2022). In our previous study, EVs from Momordica charantia possessed the potential for treating cerebral ischemia/reperfusion injury and heart disease caused by radiation (Cai et al., 2022). The EVs from black Maca have been proved for their antidepressant effects in mice (Hong et al., 2023). Here, this study isolated EVs from CB and examined their role on alleviating cognitive impairment and pathological damage in AD. CB-EVs treatment upregulated the expression of synaptic markers PSD-95 and synaptophysin, suggesting an improvement in synaptic integrity related with behavioral enhance observed in the treated AD mice.

In this study, we treated AD mice and Aβ damaged neurons with CB-EVs at different concentrations (0, 10, 20 mg/kg *in vivo*; 0, 1, 10, 100 μg/ml *in vitro*), and the results showed that a concentration of 10 mg/kg achieved significant improvement in the lesions of AD mice *in vivo*, and further improvement was not very significant at a concentration of 20 mg/kg. A likely explanation might be that 10 mg/kg was already sufficient to produce the protective effect detectable under the present experimental conditions, and increasing the dose from 10 to 20 mg/kg did not provide a clear additional benefit within the tested dose range. This interpretation was supported by the *in vitro* results that at the cellular level, research found that both 1 and 10 μg/ml CB-EVs had the best protective effect, while a concentration of 100 μg/ml had a damaging effect. Moreover, to eliminate non-specific effects, Aβ damaged neurons were treated with heat-inactivated CB-EVs for cell viability testing, and the data in Figure 5B proved that intact CB-EVs as well as internal bioactive components were essential for neuroprotective effect. Regrettably, this study did not include the heat-inactivated CB-EVs as a control in all *in vivo* and *in vitro* experiments, which prevented rigorous demonstration of the specific effect of CB-EVs.

Astrocytes and microglia, the main cells residing in the central nervous system, have become pivotal in research on neurodegenerative diseases, particularly AD. Glial cells are essential for maintaining the brain’s dynamic equilibrium through neurotransmitter uptake and circulation, inflammation management, synaptic activity regulation, ion balance, and blood-brain barrier preservation (Shah et al., 2022; Verkhratsky and Nedergaard, 2018). Growing evidence suggests that AD pathogenesis involves not only neurons but also a strong association with brain glial cells (Fakhoury, 2018). Glial cell activation can precede Aβ deposition in the early stages of AD. Our findings revealed a notable increase in GFAP astrocytes and IBA-1 microglia in 3xTg-AD mice, which was mitigated by CB-EVs treatment.

In AD patients, the rise of oxidative stress in brain is intricately linked to numerous pathological features. This link is primarily due to three factors: (a) the disruption of brain transition metal homeostasis, leading to increased binding of these metals with Aβ to form reactive species (Ganguly et al., 2017). (b) the activation and overexpression of oxidases like NADPH oxidases and monoamine oxidase B (MAO-B) (Ganguly et al., 2021). (c) Mitochondrial dysfunction (Song et al., 2021). Various *in vitro* and *in vivo* studies have shown that the Aβ peptide directly elevates reactive ROS levels, leading to oxidative stress (Butterfield et al., 2010; Cheignon et al., 2018). NUPR1, a stress-related gene, is involved in neuronal autophagy primarily by inhibiting p-mTOR activity (Xu et al., 2017), and is recognized as a pro-survival gene associated with endoplasmic reticulum stress. Recent research has pinpointed NUPR1 as an important indicator for the advancement of AD (Huang et al., 2024). Our research showed that NUPR1 was highly expressed in the brains of 3xTg-AD mice and in neurons damaged by Aβ, linked to increased levels of CHOP, MDA, and ROS. Delightingly, CB-EVs were proved to exert a significant suppressive role against oxidative stress activation in AD. In the future, we will focus on the following research: (1) include both sexes to determine the broader applicability of the findings based on the sex-specific AD trajectories; (2) the exact mechanism by which CB-EVs regulate NUPR1 and clarify its association with the antioxidant therapeutic effect.

## Conclusion

This study demonstrated that CB-EVs notably improved cognitive and exploratory deficits in 3xTg-AD mice. Long-term CB-EVs treatment reduced Aβ and p-Tau accumulation, alleviated pathological damage, protected hippocampal neurons from Aβ toxicity and oxidative stress, suggesting a potential molecular mechanism for CB-EVs in AD treatment.

## Data Availability

The original contributions presented in this study are included in the article/supplementary materials, further inquiries can be directed to the corresponding author.

## Footnotes

1
www.majorbio.com
